# Tailored NaCl Doping of PEDOT:PSS as a Hole Transport Layer for Flexible Air-Blade-Coated Devices

**DOI:** 10.1021/acsomega.5c09602

**Published:** 2026-01-23

**Authors:** Davi Emanuel Silva Monteiro, João Paulo Araújo Souza, Kaike Rosivan Maia Pacheco, Marcelo Lopes Pereira Junior, Marlus Koehler, Lucimara S. Roman, Diego Bagnis, Luana Wouk

**Affiliations:** † 28127 University of Brasília, Institute of Physics, Federal District, Brasília 70910-900, Brazil; ‡ University of Brasília, UnB Planaltina College, Graduate Program in Materials Science, Federal District, Planaltina 73345-010, Brazil; § 28122 Federal University of Paraná, Department of Physics, Curitiba, Paraná 81530-000, Brazil; ∥ Oninn Innovation Center, Organic Hybrid Printer Electronics RD, Belo Horizonte, Minas Gerais 31035-536, Brazil

## Abstract

Organic photovoltaic devices hold significant promise for sustainable energy generation, due to their low manufacturing cost and benefits such as lightness, semitransparency, and flexibility. This study explores the experimental and theoretical study of the effects of sodium chloride (NaCl) doping on PEDOT:PSS, with a focus on its photovoltaic properties in inverted all air blade-coated devices. By varying NaCl concentrations, we analyze key performance metrics and annealing treatment, open-circuit voltage, short-circuit current density, fill factor, and power conversion efficiency. The results indicate that lower concentrations of NaCl substantially improve these parameters, while higher concentrations can impair the efficiency of the device. Post annealing was found to improve photovoltaic performance, especially in samples with low NaCl concentrations. Advanced characterization techniques, including atomic force microscopy, Raman spectroscopy, and scanning electron microscopy, revealed that doping with NaCl improves the molecular organization of PEDOT:PSS, leading to better light transmission and energy efficiency. Theoretical results indicate that NaCl interacts with the thiophene rings of PEDOT:PSS, modifying its electronic and structural properties. These results highlight the fundamental role of doping and processing conditions in optimizing the performance of organic solar cells, providing valuable information for the development of efficient, economical, and sustainable photovoltaic technologies.

## Graphical abstract

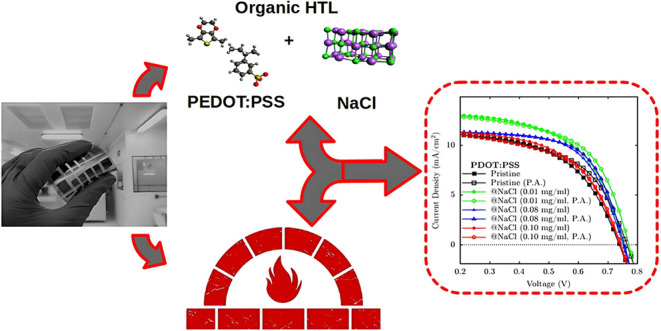

## 1 Introduction

Organic solar cells (OSCs) stand out as one of the most promising technologies in the new generation of photovoltaic devices, thanks to their lightweight design, economical solution-based processing, flexibility^1–4^ and low-cost manufacturing.^5,6^ In addition, they present significant advantages for global applications, such as in wearable electronic devices and building-integrated photovoltaics, overcoming the limitations of conventional technologies based on crystalline silicon (C–Si).^7^ Their versatility for use indoors and their adaptability to innovative designs reinforce their potential as ideal candidates for integration into a wide range of architectural and functional contexts.^8,9^

To increase the power conversion efficiency (PCE), a comprehensive understanding of the photophysical properties is crucial for the development of new materials and the scaling of organic solar cells (OSCs), from laboratory prototypes to industrial manufacturing processes. Among the various parameters considered in OSC design, one that has driven significant advances is the architecture of the device. The most traditional configuration (considering the use of interfacial layers), known as the regular architecture, features a transparent anode coated with a hole transport layer (HTL), followed by the active layer (AL), an electron transport layer (ETL), and finally the metallic cathode. Inverted OSC architectures, on the other hand, generally consist of the following sequence of layers: cathode, ETL, AL, HTL, and anode.^10^ Each of these layers plays a specific role in the device’s efficiency, with the active layer receiving the greatest attention.

In this context, the active layer has attracted the most interest, with extensive research focused on its molecular structure and doping processes.^11–13^ By 2024, for instance, a network of more than 2,980 studies would be concentrated on the active layer, in contrast to only 793 studies focused on interfacial layers. Despite receiving less attention, these intermediate layers are essential for facilitating efficient charge extraction, mitigating energy losses, and ensuring optimal contact between the electrodes and the organic active layer.

Besides, advances in active layer efficiency require modifications to the chemical structure of the material, generating a change in the energy levels, morphology and fabrication methods of OSCs.^14–17^ As a result, many of the existing intermediate layers are no longer compatible with the new active layer materials, creating challenges to further improve the efficiency and stability of the OSC. For inverted OSCs, oxide-derived materials, such as MoO_3_, V_2_O_5_, WO_3_ and NiO, are widely studied as HTLs.^18^ Although oxide materials increase device stability, their sol–gel coating processes require high temperatures, which can cause damage and induce undesirable morphological changes in the device structure. Nanoparticle-based structures have also shown potential, although obtaining uniform and continuous morphologies remains a significant challenge.

Polymer-based HTLs, therefore, remain the most viable option for transitioning OSCs from laboratory to scalable manufacturing. Among these, PEDOT:PSS (poly­(3,4-ethylenedioxythiophene):polystyrenesulfonate) stands out as the most commonly employed HTL.^19,20^ Efforts to improve PEDOT:PSS performance have explored variations in its composition, doping strategies, and thermal treatments.^21,22^ Doping agents for PEDOT:PSS, including alcohols, oxides, and amines, alter the interaction between PEDOT and PSS, consequently modulating carrier mobility and energy levels depending on the dopant’s properties.^22^ However, few studies have addressed its role in inverted OSC architectures, particularly for fully flexible devices coated with blades processed under ambient air conditions.

The doping of PEDOT:PSS with inorganic salts has emerged as an effective approach to optimize its electrical and morphological properties by modifying the interaction between the PEDOT and PSS chains. Recent studies have shown that additives such as zinc iodide *ZnI*_2_ ^23^ and sodium sulfite *Na*_2_ *SO*_3_ ^24^ promote structural reorganization of the film, partial removal of the insulating PSS fraction, and improved charge transport, resulting in significant performance gains in optoelectronic devices.

In this study, we examine the effects of sodium chloride (NaCl) doping on the electrical properties of PEDOT:PSS, focusing on key photovoltaic parameters such as open-circuit voltage (*V*_OC_), short-circuit current density (*J*_SC_), fill factor (FF), and power conversion efficiency (PCE), see Figure 1. Additionally, we investigate the impact of annealing and post annealing on optimizing device performance, providing insights into the interplay between doping concentration and thermal treatment in achieving enhanced device efficiency and stability.

**Figure 1:**
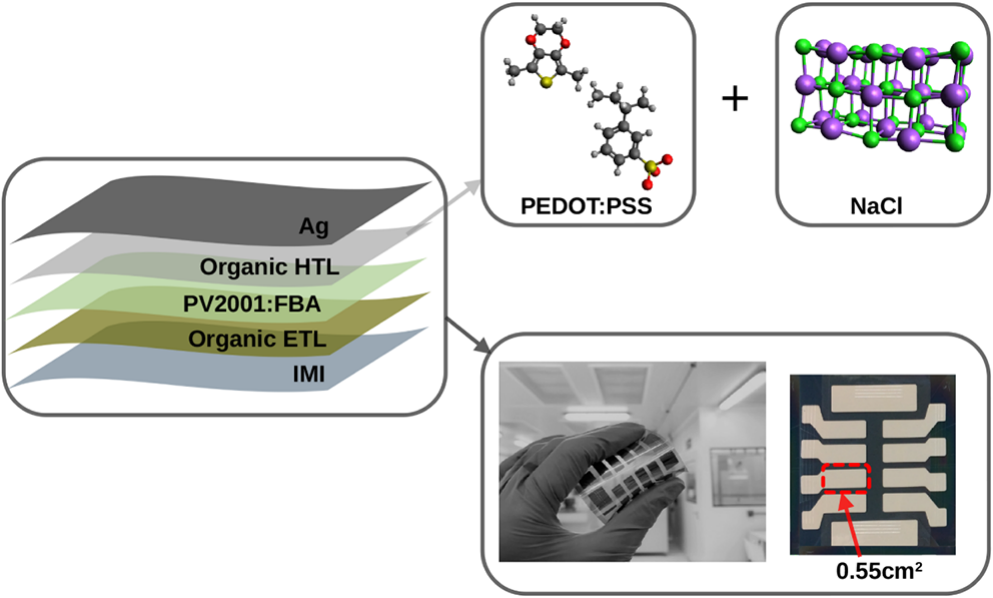
Schematic representation of the architecture of an organic solar cell (OSC), which consists of the following layers: IMI (ITO/Ag/ITO), ETL, active mixture PV2001:Fullerene-Based Aceptor (FBA), HTL and the top silver electrode (Ag). On the right, the molecular structures of PEDOT:PSS and NaCl used in the intermediate HTL layer and the flexible device with an area of 0.55 cm^2^ are highlighted.

## 2 Experimental Procedures

The devices were processed in ambient atmosphere by blade coating using an Erichsen Coatmaster 500, following the methodology previously described by Miranda et al.^25^ A polyethylene terephthalate (PET) substrate covered with a sputtered multilayer of indium tin oxide/silver/indium oxide (ITO/Ag/ITO, or IMI) with a sheet resistance of 10Ω/sq and transmittance of 88% at 570 nm (Eastman) was used. The PET/IMI substrates, with an area of 2500 mm^2^, were patterned by *CO*_2_ laser etching.

An electron transport layer (ETL) of polyethylenimine (PEI) was deposited with a gap of 575 μm between the blade and the substrate at a speed of 5 mm/s, followed by annealing at 100^◦^C. The active layer (AL) consisted of a mixture of a low bandgap copolymer (1.67 eV, Raynergy Tek) with the acceptor PC_60_BM (Nano-C), dissolved in anhydrous o-xylene. A PEDOT:PSS film (CLEVIOS HTL Solar, Heraeus) was deposited on the AL, acting as a hole transport layer (HTL). The solution was previously filtered to remove solid impurities. The top electrode consisted of a 200 nm layer of silver (Ag), deposited via thermal evaporation using the Angstrom Engineering NexDep 400 system. The use of an evaporation mask allowed the definition of eight devices per substrate, each with an active area of 0.55 cm^2^.^25^

In this study, different concentrations of NaCl were incorporated into the PEDOT:PSS solution (see Table 1) to investigate possible performance improvements. The samples also underwent a postannealing (P.A.) process at 140°C, 150°C, and 160°C, with the aim of optimizing the electrical response of the devices. The preparation requires small amounts of NaCl, so the NaCl was first dissolved in isopropyl alcohol (IPA) and then added to the PEDOT:PSS solution. The mixtures were stirred for 15 min to promote homogeneity before deposition. To isolate the effect of IPA, the control sample was diluted in the same proportion.

**Table 1:** Final Concentration of the PEDOT:PSS Mixture with NaCl

sample	NaCl Concentration (mg/mL)
PEDOT:PSS	Without NaCl
PEDOT:PSS	0.01
PEDOT:PSS	0.08
PEDOT:PSS	0.10

Transmittance measurements were performed using a UV–vis spectrometer (Shimadzu 2600 UV Vis Spectrophotometer) in the wavelength range of 200 to 1000 nm. Morphological evaluations were performed using atomic force microscopy (AFM) in intermittent mode (Shimadzu SPM-9700). AFM images were obtained on films with the same structure used to fabricate the devices. The scanning electron microscope used was the FEI Quanta 450 FEG.

A Class AAA solar simulator (Wacom WXS-156S-10), incorporating a xenon light source and an AM 1.5G spectrum filter, was used to acquire current–voltage (*J*-*V*) density curves under standard 1 sun conditions. Raman spectra were obtained using a Renishaw 3000 Raman Imaging Microscopy System. The laser excitation line used was He–Ne (632.8 nm). The measurement was performed using a Keithley peak ammeter with power supply (model 6487), a monochromator/spectrometer (1/4 m Oriel), and illumination power from a 150 W Oriel xenon lamp.

## 3 Results and Discussions

### 3.1 Device Optimization (NaCl Concentration)

After the fabrication and characterization of the devices, the first step of the analysis consisted in evaluating the electrical parameters obtained from the current density versus voltage (J-V) curves under illumination, considering both the shape of the curves and their variations as a function of the applied fabrication conditions. In this context, Figure 2 presents the characteristic current density versus voltage curves for samples containing pristine PEDOT:PSS, as well as samples doped with different concentrations of NaCl and subjected to postannealing (P.A.) treatment.

**Figure 2:**
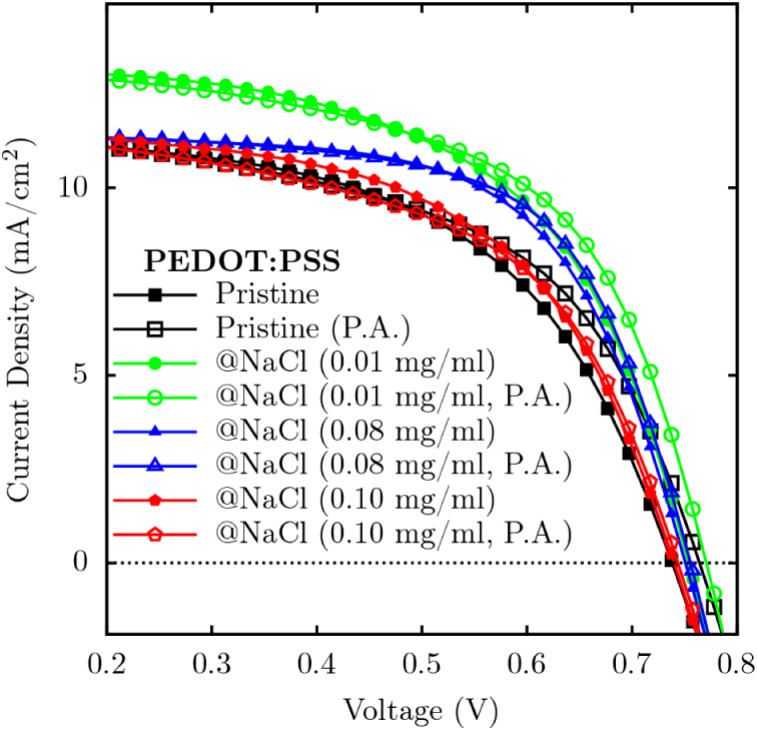
*J*-*V* curves for PEDOT:PSS samples under varying conditions: pristine, doped with NaCl at concentrations of 0.01, 0.08, and 0.10 mg/mL, and with or without postannealing treatment.

At low concentrations, doping with NaCl improves device parameters such as *V*_OC_ and *J*_SC_, see Table 2. Doping with 0.01 mg/mL NaCl results in a 1.14% increase in PCE, accompanied by simultaneous improvements in *V*_OC_, *J*_SC_, FF and PCE. However, higher NaCl concentrations, such as 0.10 mg/mL, negatively affect PCE, reducing the overall efficiency of the device by 0.42%, despite an increase in *J*_SC_.

**Table 2:** Device Performance Parameters (*V*_OC_, *J*_SC_, FF, PCE, *R*_s_, *R*_sh_) for PEDOT:PSS Samples under Varying Conditions: Pristine, Doped with NaCl (0.01, 0.08, and 0.10 mg/ml), and with or without Post-Annealing (P.A.) at 140°C

Sample	*V*_OC_ (V)	*J*_SC_ (mA/cm^2^)	FF (%)	PCE (%)	*R*_s_ (Ω)	*R*_sh_ (Ω)
PEDOT:PSS (Reference)	0.73 ± 0.02	11.46 ± 0.50	55.46 ± 3.00	4.69 ± 0.50	27 ± 3	1086 ± 500
PEDOT:PSS (P.A.)	0.76	11.50	55.42	4.89	26	984
PEDOT:PSS@NaCl (0.01 mg/mL)	0.75 ± 0.02	13.21 ± 0.50	58.46 ± 3.00	5.83 ± 0.50	22 ± 3	2260 ± 500
PEDOT:PSS@NaCl (P.A.) (0.01 mg/mL)	0.77	13.17	59.00	6.04	21	1510
PEDOT:PSS@NaCl (0.08 mg/mL)	0.75 ± 0.10	11.41 ± 2.00	65.00 ± 4.00	5.58 ± 0.50	23 ± 10	3762 ± 500
PEDOT:PSS@NaCl (P.A.) (0.08 mg/mL)	0.75	11.49	65.44	5.70	22	2913
PEDOT:PSS@NaCl (0.10 mg/mL)	0.72 ± 0.40	11.98 ± 4.00	49.31 ± 15.00	4.27 ± 3.00	28 ± 20	506 ± 2000

Postannealing treatment enhances the positive effects of NaCl doping in all samples. In the case of PEDOT:PSS P.A. 0.01 mg/mL, annealing increases the PCE by 0.21%, while the values of *V*_OC_, *J*_SC_ and FF show increases of 0.017 V, 0.04 *mA*/*cm* ^2^ and 0.04%, respectively, compared to samples without annealing.

Compared to the reference sample, the NaCl-doped sample benefits from annealing, especially at a concentration of 0.01 mg/mL, improvement in PCE of 1.35%. Other samples also showed improvements, but not as significant as the 0.01 mg/mL sample. The *R*_s_ values without heat treatment are lower for PEDOT:PSS doped with 0.01 mg/mL NaCl. Postannealing reduces the *R*_s_ even more in the samples with 0.01 and 0.08 mg/mL NaCl. This effect minimizes ohmic losses, increasing the short-circuit current, which in turn improves the FF and raises the overall efficiency of the device. The *R*_sh_ is higher for all samples doped with NaCl, except for the 0.10 mg/mL concentration, indicating a reduction in leakage current losses. This improves *V*_*oc*_, increases the solar cell’s efficiency, and its stability.

To understand the observed improvements, theoretical calculations were performed via DFT to analyze the interactions between NaCl and the PEDOT:PSS oligomer, designated Configuration 1 and Configuration 2 (see Figure 3). The interaction with the chloride ion (Cl^–^) led to an increase in the molecular length of PEDOT by 0.27 Å in Configuration 1 and 0.35 Å in Configuration 2, resulting in a planar molecular conformation (see Supporting Information S2). This increased planarity, due to the local charge redistribution induced by the dopant ion, facilitates the formation of polarons^26^ or bipolarons.^27^ These species are characterized by localized charge carriers coupled to lattice distortions, which enhance charge transport through the polymer matrix.

**Figure 3:**
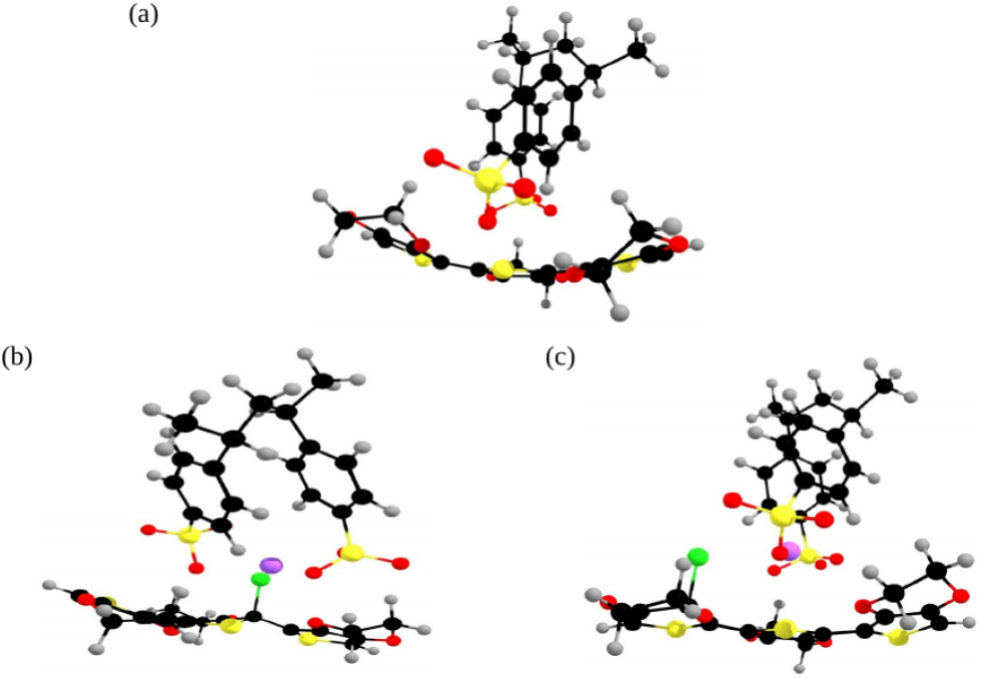
Illustration of (a) the PEDOT:PSS structure and its interaction with NaCl in two distinct cases: (b) Configuration 1, where NaCl is positioned closest to PEDOT, and (c) Configuration 2, where NaCl is positioned closest to PSS.

The interaction between Cl and PEDOT:PSS also results in changes in the overlap of molecular orbitals, increasing π–π stacking interactions and promoting better charge delocalization, see Supporting Information S3.

Furthermore, the enhanced planarity may also promote better molecular stacking, contributing to increased crystallinity of the films. This improved crystallinity, combined with changes in the overlap of molecular orbitals caused by the interaction between Cl and PEDOT:PSS, strengthens π–π stacking interactions and promotes more efficient charge delocalization (see Supporting Information S3). These effects can enhance the conductivity of the films and positively impact key device parameters, such as *J*_*SC*_, as observed in Figure 2. Configurations 1 and 2 show lower total energy and a reduction in Gibbs free energy variation, see Supporting Information S4, suggesting greater thermodynamic stability. The improvement observed with the lower NaCl concentration can be explained by the formation of more efficient charge paths and the reduction of recombination losses in the NaCl-doped PEDOT:PSS films. On the other hand, higher concentrations have a negative effect on the device due to excess NaCl, increasing resistance and leading to higher recombination rates.

Figure 4 shows the Raman spectrum for PEDOT:PSS with NaCl concentrations of 0.01, 0.08, and 0.10 mg/mL. The Raman spectroscopy was applied to analyze the interaction between PEDOT:PSS and NaCl, to investigate possible molecular changes in the polymer’s structure. The Raman spectrum, presented at Figure 4, revealed changes in the vibrational bands around 1430 cm^–1^ and 1600 cm^–1^, commonly attributed to symmetric and asymmetric deformations of the C_α_=C_β_ ^28^ bonds in the thiophene ring,^22^ characteristics of the PEDOT conjugated structure.^29–31^ In addition, ring stretching vibrations, corresponding to the C_α_–C_α_ and C_β_=C_β_ bonds, are observed at 1257 and 1365 cm^–1^, respectively.^32^

**Figure 4:**
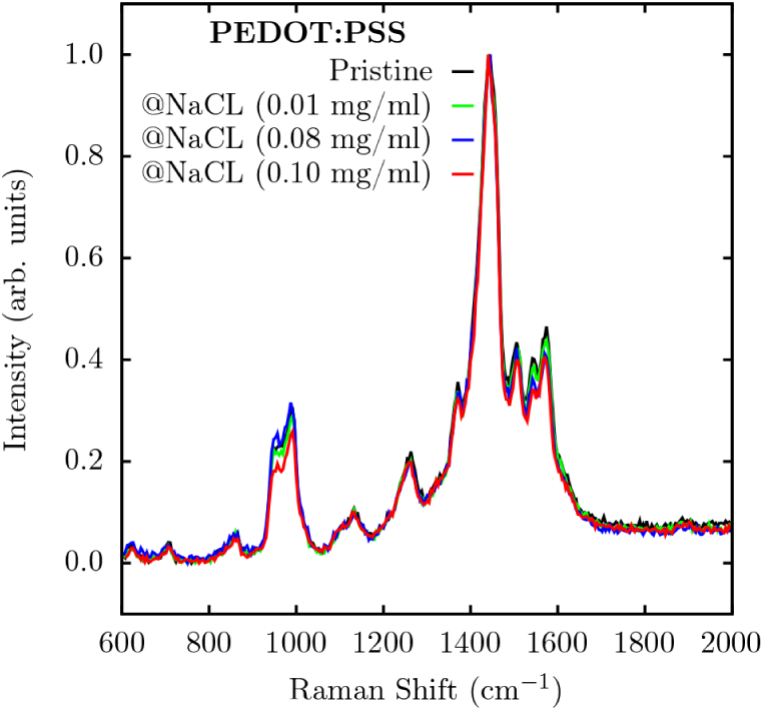
Experimental Raman spectroscopy of pure PEDOT:PSS and samples with varying NaCl concentrations.

All the samples show a shift in the 1500 cm^–1^ region, attributed to the vibrations of carbon double bonds (C=C), due to the interaction with NaCl. However, the sample with the lowest concentration of NaCl shows a red shift, indicating that the vibrations are becoming less energetic. This result can be attributed to the increase in the length of the C=C bond (see Supporting Information S2–S4), suggesting a conformational change to a more stable molecular geometry. This conformational change is related to a transition from the benzenoid form to the quinoid form, which is associated with better electrical conduction.^22^ On the other hand, increasing the concentration of NaCl leads to a blue shift, indicating more energetic vibrations.

Figure 5 illustrates the variation in the transmittance of PEDOT:PSS under different NaCl concentrations. All curves show high transmittance in the region of 400 to 500 nm, with samples treated with NaCl showing even higher transmittance at 500 nm. Above 600 nm, samples with concentrations of 0.01 and 0.08 mg/mL show low transmittance, which indicates greater optical absorption and, consequently, an increase in the photocurrent generated by the device. This behavior corroborates the fact that these samples have low *R*_*s*_ and high *R*_*sh*_, favoring efficient carrier extraction and resulting in greater device efficiency. On the other hand, the curve corresponding to the concentration of 0.10 mg/mL (red) shows an increase in transmittance above 600 nm, due to the influence of the high concentration of NaCl.

**Figure 5:**
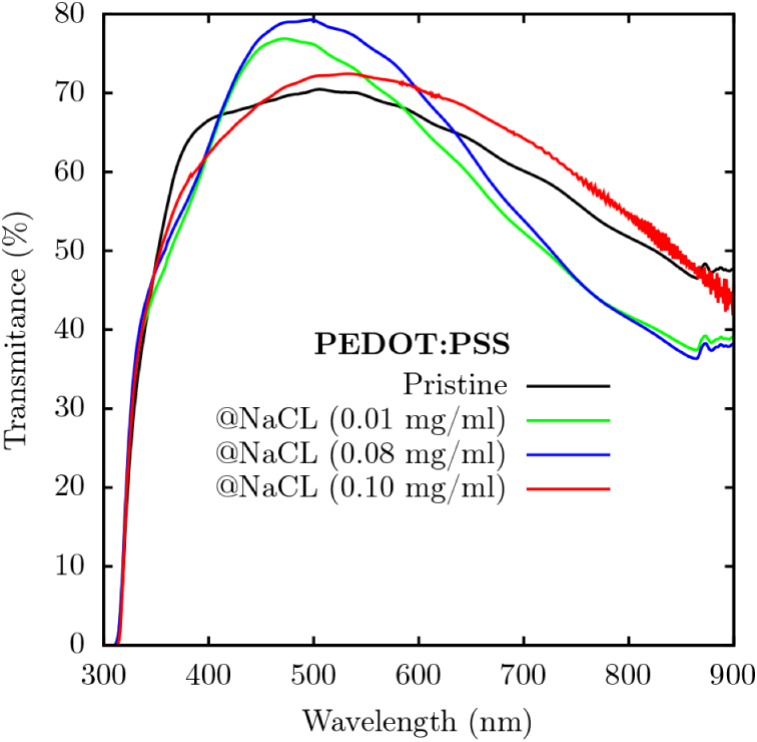
Transmittance (%) versus wavelength (nm) for pure PEDOT:PSS and samples with different NaCl concentrations: 0.01 mg/mL, 0.08 mg/mL, and 0.10 mg/mL.

To further investigate the influence of NaCl on the optical properties of the PEDOT:PSS films, transmittance measurements were performed on samples prepared with different NaCl concentrations. Figure 5 presents the corresponding transmittance spectra. All samples exhibited high transmittance in the 400–500 nm range, with the NaCl-treated films showing even higher transmittance around 500 nm. In contrast, above 600 nm, the samples doped with 0.01 and 0.08 mg/mL displayed lower transmittance, suggesting enhanced optical absorption, which is a factor that can contribute to increased photocurrent generation in devices. This behavior aligns with the electrical performance of these samples, which showed low *R*_*s*_ and high *R*_*sh*_ values, favoring efficient charge extraction and improved device efficiency.

On the other hand, the sample with 0.10 mg/mL NaCl (red curve) exhibited higher transmittance above 600 nm, likely due to the effects associated with the higher salt concentration.

The increase in transmittance in the shorter wavelength regions is associated with changes in the film morphology caused by the interaction of Na^+^ ions with the sulfonate $\left({\mathrm{SO}}_{3}^{-}\right)$ groups of PSS. This interaction promotes more efficient phase separation and stimulates the molecular reorganization of PEDOT:PSS into a denser, more homogeneous, and less opaque structure. A structural rearrangement can contribute to improvements in both the electrical conductivity and the optical properties of the material. This effect suggests a change in the polymer organization, as evidenced by Raman spectroscopy, which promotes greater charge delocalization and consequently reduces device efficiency.

This effect suggests a change in the polymer organization, as evidenced by Raman spectroscopy, which promotes greater charge delocalization and is associated with shifts in the vibrational bands attributed to the symmetric and asymmetric deformations of the C=C bonds in the thiophene ring. For the sample with 0.01 mg/mL of NaCl, a shift to lower frequencies (red shift) is observed, indicating less energetic vibrations and increased structural flexibility. This effect implies a decrease in the rigidity of the vibrational modes, which may favor absorption in the near-infrared region (above 700 nm), where a reduction in transmittance is observed. Therefore, this reduction can be interpreted not only as a result of morphological reorganization but also as a spectral signature of the softening of vibrational modes induced by low-concentration doping. The Figure 6 shows Atomic Force Microscopy (AFM) and Scanning Electron Microscopy (SEM) images of pure PEDOT:PSS films doped with different concentrations of NaCl: 0.01 mg/mL, 0.08 and 0.10 mg/mL.

**Figure 6:**
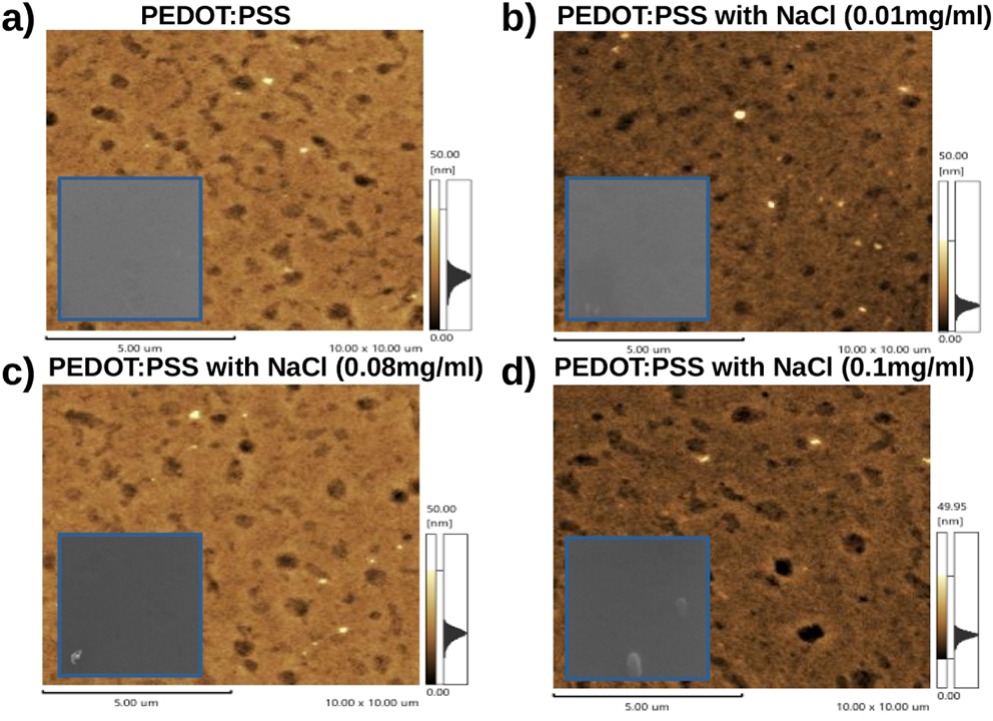
Atomic Force Microscopy and Scanning Electron Microscopy of PEDOT:PSS films: (a) pure PEDOT:PSS, (b) PEDOT:PSS doped with 0.01 mg/mL NaCl, (c) PEDOT:PSS doped with 0.08 mg/mL NaCl and (d) PEDOT:PSS doped with 0.10 mg/mL NaCl.

Figure 6a shows the surface morphology of pure PEDOT:PSS, where a relatively homogeneous surface is observed, with few PEDOT domains, indicating a balance between the dispersion of the PEDOT and PSS phases. In contrast, Figure 6b shows PEDOT:PSS doped with 0.01 mg/mL of NaCl, revealing an evident reorganization of the film morphology. The surface now exhibits a more pronounced texture, with larger domains, but without relevant defects or discontinuities. This change is attributed to the suppression of electrostatic interactions between the PSS chains by Na^+^ ions, promoting partial segregation of PEDOT on the surface and reorientation of the chains, which favors the formation of more efficient conductive networks.^22^ As a result, this sample showed the best electrical performance among all concentrations evaluated. This behavior is in accordance with mechanisms described in the literature, which indicate that the introduction of salts or dopants aids in the reorganization of the polymer network, reducing the potential barrier for charge transport.^33^

The Figure 6c,d illustrate the morphologies of PEDOT:PSS doped with 0.08 and 0.10 mg/mL of NaCl, respectively, showing more heterogeneous surfaces. At a concentration of 0.08 mg/mL, regions with well-defined pores and aggregates are observed. Although the conductive domains of PEDOT are still present, their distribution becomes more irregular and discontinuous. Dark and bright spots indicate abrupt variations in topography and a more dispersed height distribution, suggesting internal stresses and possible local collapse of the structure, probably due to excessive PEDOT segregation and Na^+^ ion accumulation. At a concentration of 0.10 mg/mL, the film morphology shows even more pronounced degradation. The images reveal depressed regions, evident pores, and irregularly distributed aggregates. The darker areas point to local collapses and the formation of microcracks, indicative of internal stresses. In addition, there is a significant reduction in the continuity of the conductive domains, compromising the electrical percolation pathways. Thus, although small amounts of Na^+^ ions improve morphology and electrical performance, excess dopant severely disrupts the structure, forming aggregates and defects that limit charge transport.^34–37^ Figure 6a–d reinforce these observations, showing that lower concentrations of NaCl favor a homogeneous morphology, while high concentrations, such as 0.10 mg/mL, result in agglomerations and greater morphological disorder. In the following section, the effect of postdeposition heat treatment, Post Annealing (P.A.) on the sample with 0.01 mg/mL will be investigated.

### 3.2 Light-Soaking Effect

To evaluate the influence of light exposure on device performance, *J*-*V* measurements were conducted on pure PEDOT:PSS and NaCl-doped PEDOT:PSS samples after different exposure times. Figure 7 shows the *J*-*V* curves for samples of pure PEDOT:PSS and PEDOT:PSS doped with NaCl, 0.01 mg/mL, tested at different light exposure intervals. The application of the light immersion effect is well documented to improve the performance of organic solar cells (OSCs), leading to higher PCE and charge carrier mobility, as demonstrated by Jeon et al.^38^ These enhancements are often linked to morphological rearrangements in the active layer or the reduction of degradation effects, as discussed by Dusza et al.^39^

**Figure 7:**
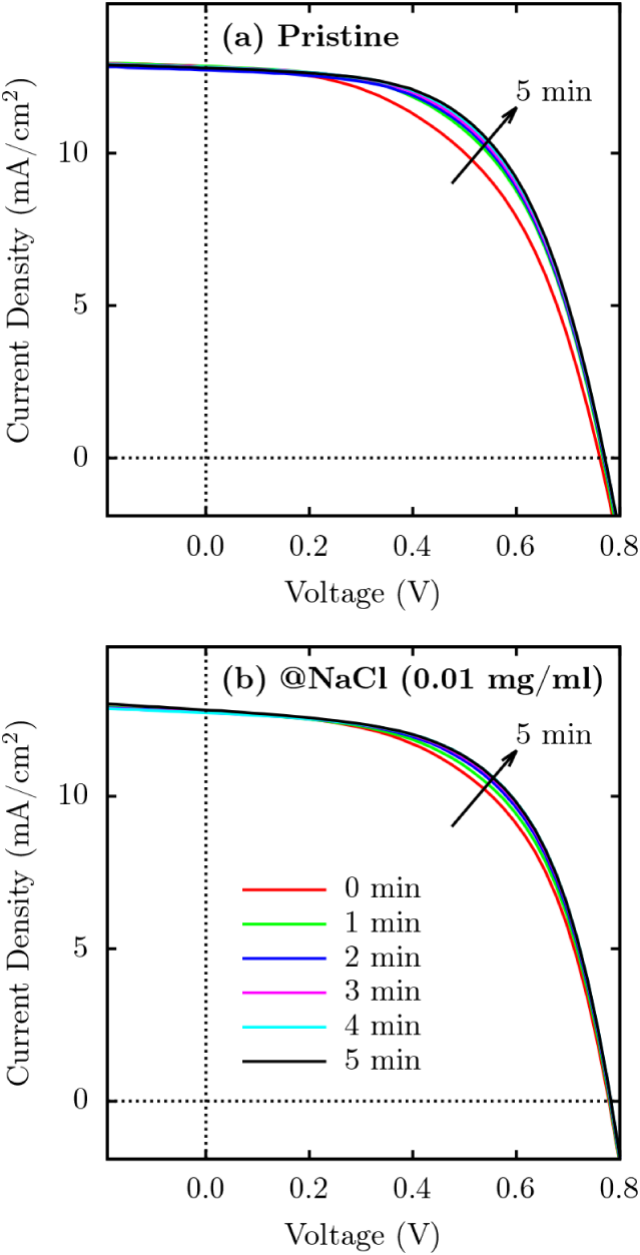
Current density (*J*) versus applied voltage (*V*) for pristine PEDOT:PSS (a) and PEDOT:PSS doped with 0.01 mg/mL of NaCl (b), subjected to varying light exposure times from 0 to 5 min.

The pure PEDOT:PSS, Figure 7a, exhibits a good response to light exposure, as evidenced by the differences in the *J*-*V* curves for each time interval. After 5 min of exposure, an improvement in current density is observed, possibly due to morphological changes or relaxation effects that enhance charge carrier transport.

On the other hand, for NaCl-doped PEDOT:PSS (0.01 mg/mL), Figure 7b, the *J*-*V* curves show minimal variation across different illumination intervals, indicating limited sensitivity of the doped material to light exposure. The curve corresponding to 4 min of exposure shows a slight increase in current density in the high voltage region, which may indicate a small improvement in efficiency and reduction in recombination losses. This behavior suggests that the addition of NaCl may contribute to greater stability or structural reorganization under illumination, slightly reducing recombination losses.

The behavior of NaCl-doped PEDOT:PSS can be attributed to the dopant’s ability to mitigate charge carrier traps more effectively than the pure material, as highlighted in previous studies.^40^ The presence of NaCl promotes improved molecular packing and reduces energetic disorder, leading to more efficient charge transport under sustained illumination. Furthermore, the light exposure process appears to induce microstructural changes in the doped material, optimizing the PEDOT:PSS–PSS interface and improving charge extraction. These findings underscore the critical role of doping and light exposure in optimizing the performance of PEDOT:PSS-based devices.

## 4 Conclusions

Doping PEDOT:PSS with NaCl, in combination with heat treatment (annealing), significantly improves photovoltaic performance. The *J*-*V* characteristic curves for low NaCl concentrations, 0.01 mg/mL, improve the main device parameters, including *V*_OC_, *J*_SC_, FF and PCE. However, higher NaCl concentrations negatively affect these parameters, highlighting the importance of optimizing the dopant concentration to obtain maximum device performance.

Theoretical studies show the interaction of Cl with the thiophene ring of PEDOT, inducing changes in the C–C and C=C bonds, leading to changes in molecular organization. These findings, supported by Raman spectroscopy analyses, highlight the relationship between molecular organization and increased PCE. This demonstrates that precise control over the composition and processing of PEDOT:PSS is crucial for the advancement of photovoltaic device technology.

Annealing proved to be particularly effective, especially for samples with low NaCl concentrations, 0.01 mg/mL, where a 1.35% increase in PCE was observed. This increase is attributed to the optimization of the electrolyte interface and the improved current density resulting from more efficient molecular organization. Analyses of microstructural and electronic properties corroborate these findings, revealing that NaCl doping and annealing synergistically improve the organization and morphology of PEDOT:PSS, promoting efficient charge transport and more homogeneous interfaces.

This study highlights the fundamental role of dopant selection and processing conditions in optimizing the performance of PEDOT:PSS-based solar cells. In addition, it provides fundamental insights into the molecular interactions and structural dynamics that govern the material’s properties. These findings lay a solid foundation for the development of next-generation photovoltaic technologies that are more efficient, economically viable and sustainable.

## Supplementary Material



## Notes

Section S1: evaluation of different performance parameters of PEDOT:PSS films; Section S2: theoretical calculation details; Section S3: analysis of chemical bond lengths, PEDOT chain length, and thiophene ring dihedral angles; Section S4: analysis of HOMO and LUMO molecular orbitals; Section S5: comparison between the electrical parameters of different samples of PEDOT:PSS doped with NaCl at various concentrations; Section S6: chemical structure of the donor polymer PV2001 (PDF)
