# Supplementary material for: Tailored NaCl Doping of PEDOT:PSS as a Hole Transport Layer for Flexible Air-Blade-Coated Devices
Source: ACS Omega. 2026 Jan 23;11(4):5733–40. doi: 10.1021/acsomega.5c09602 (PMC12878779; doi:10.1021/acsomega.5c09602)
Supplement: Supplementary file 1 [file ao5c09602_si_001.pdf]

# – Supporting Information –
Tailored NaCl Doping of PEDOT:PSS as a Hole Transport Layer for Flexible Air-Blade-Coated Devices

Davi Emanuel Silva Monteiro,[†,‡] João Paulo Araújo Souza,[*,†] Kaike Rosivan Maia Pacheco,[¶] Marcelo Lopes Pereira Junior,[‡] Marlus Koehler,[¶] Lucimara Stolz Roman,[¶] Diego Bagnis,[§] and Luana Wouk[*,†]

†*University of Brasília, Institute of Physics, Brasília, Federal District, BR, 70910-900.*

‡*University of Brasília, UnB Planaltina College, Graduate Program in Materials Science, Planaltina, Federal District, BR, 73345-010.*

¶*Federal University of Paraná, Department of Physics, Curitiba, Paraná, BR, 81530-000.*

§*Oninn Innovation Center, Organic Hybrid Printer Electronics RD, Belo Horizonte, Minas Gerais, BR, 31035-536.*

E-mail: joaojpkl56@gmail.com; luana.wouk@unb.br

Contents

S1 Evaluation of different performance parameters of PEDOT:PSS films treated with NaCl at three different heat treatment temperatures: 140 °C, 150 °C and 160 °C. (Annealing treatment) **S3**

S2 Theoretical Calculation Details **S7**

S3 Analysis of Chemical Bond Lengths, PEDOT Chain Length, and Thiophene Ring Dihedral Angles **S8**

S4 Analysis of HOMO and LUMO Molecular Orbitals **S11**

S5 Comparison between the electrical parameters of different samples of PEDOT:PSS doped with NaCl at various concentrations **S12**

S6 Chemical structure of the donor polymer PV2001 **S13**

References **S14**

S1 Evaluation of different performance parameters of PEDOT:PSS films treated with NaCl at three different heat treatment temperatures: 140 °C, 150 °C and 160 °C. (Annealing treatment)

we investigate the influence of annealing at different temperatures on the performance of PEDOT:PSS. Figure S1 and Figure S2 shows the J-V curves for samples annealed at 140 °C, 150 °C, and 160 °C. The Figure S1 shows statistics for 24 devices.

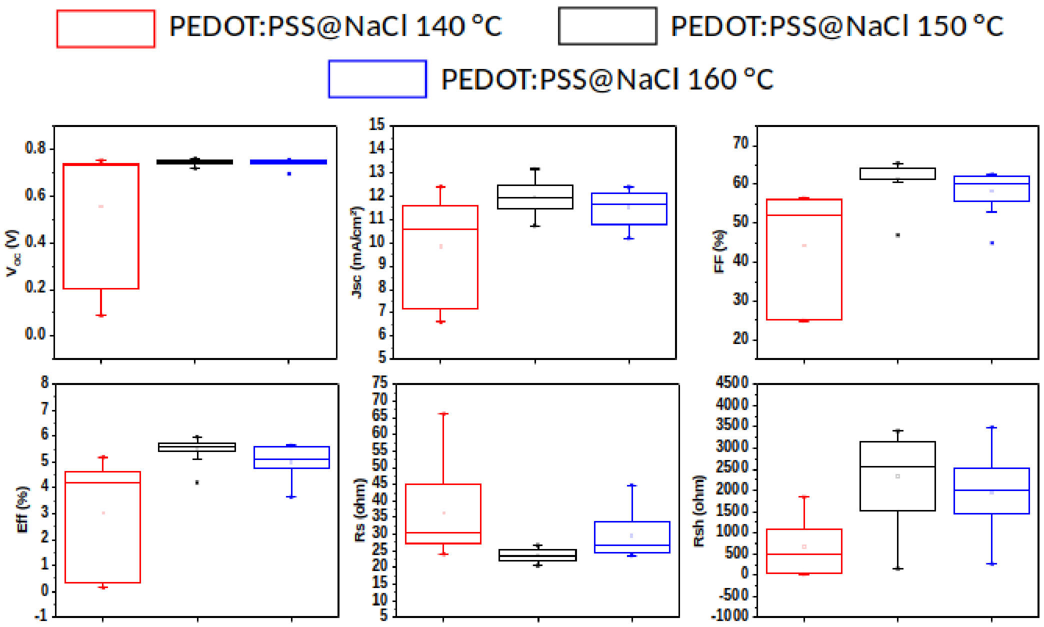

Figure S1: Photovoltaic performance parameters of devices based on PEDOT:PSS treated with NaCl at different annealing temperatures (140 °C, 150 °C, and 160 °C). The analyzed parameters include open-circuit voltage (V_{OC}), short-circuit current density (J_{SC}), fill factor (FF), conversion efficiency (Eff), series resistance (Rs), and shunt resistance (Rsh).

The J-V curves exhibit similar behaviors, indicating that J_{SC} remains relatively consistent across the different annealing temperatures. However, annealing at 150 °C improves the device performance, evidenced by the increased fill factor (FF) and power conversion efficiency (PCE), detailed in Table S1. Thermal annealing is widely recognized as a process for improv-

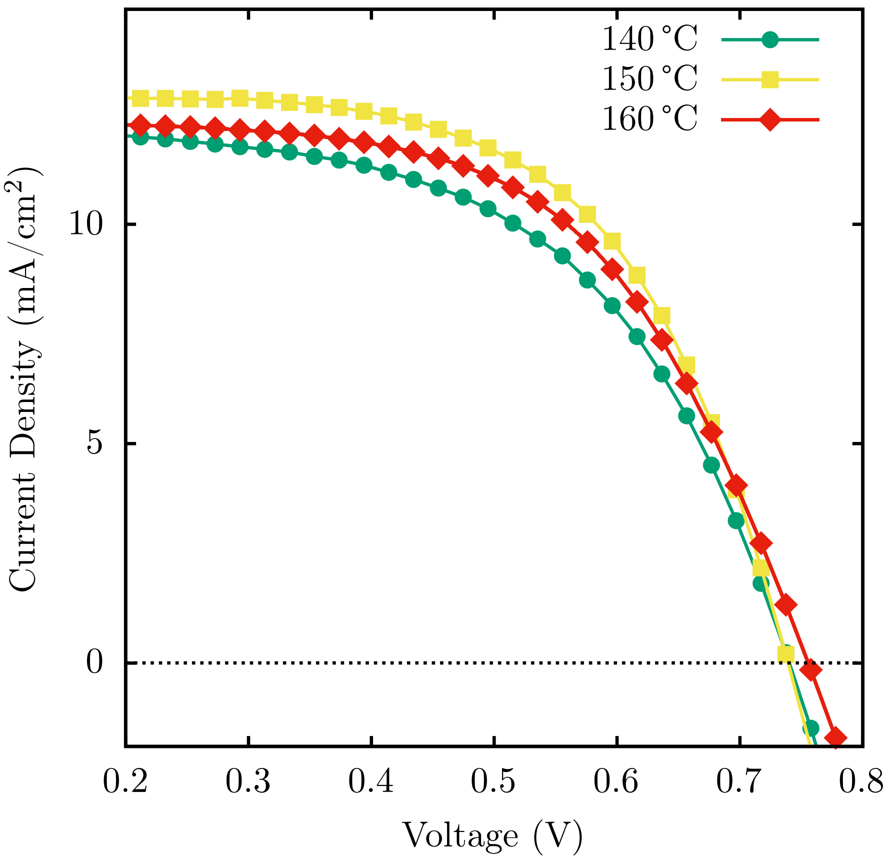

Figure S2: Current density (J) as a function of applied voltage (V) for a PEDOT:PSS, annealed at different temperatures 140 °C (green), 150 °C (yellow), and 160 °C(red)

ing device performance by promoting phase separation, enhancing molecular organization, increasing charge mobility, and reducing energetic disorder.[1–6] Candan[7] demonstrated that annealing at 150 °C improves charge separation and carrier mobility in P3HT:PCBM photoactive layers. In contrast, Shahidul Alam et al.[8] reported that annealing enhances phase separation and reduces recombination losses, leading to higher device efficiencies.

As the applied voltage increases, the J-V curves indicating that V_{OC} is affected by variations in annealing temperature. However, the curve corresponding to 150 °C shows a slightly larger area under the curve, due to the increase in J_{SC}, as detailed in Table S1. The FF also improves at 150 °C, optimizing the overall performance of the device at this temperature. At higher voltages, particularly between 0.6 V and 0.8 V, all the curves exhibit a rapid drop

Table S1: Device performance parameters (V_{OC}, J_{SC}, FF, PCE, R_s, R_{sh}) for PEDOT:PSS samples under varying conditions: pristine, doped with NaCl (0.01, 0.08, and 0.10 mg/ml), and with or without post-annealing (P.A.) at 140 °C. The devices are fabricated in air and compared with the same batch of experiments. (They are manufactured on the same day with the same relative humidity.)

Annealing Temperature	V_{OC} (V)	J_{SC} (mA/cm^2)	FF (%)	PCE (%)	R_s (Ω)	R_{sh} (Ω)	AL Thickness (nm)
140 °C	0.74 ± 0.10	12.41 ± 2.00	56.42 ± 5.00	5.18 ± 1.00	25 ± 10	1017 ± 500	285
150 °C	0.73 ± 0.02	11.96 ± 0.50	61.32 ± 3.00	5.97 ± 0.50	20 ± 3	1477 ± 500	285
160 °C	0.75 ± 0.03	12.39 ± 1.10	60.06 ± 3.00	5.63 ± 0.50	28 ± 5	3493 ± 500	285

in current density, a typical behavior as the device approaches its operational voltage limit typical diode-like behavior. These results indicate that, although the PCE reaches a maximum value at 150 °C, further increases in annealing temperature result in a slight reduction in FF and PCE. As shown in Table S1, the PCE increases from 140 °C to 150°C, reaching the highest value of 5.97% at 150 °C. This behavior can be attributed to thermal stress, which introduces defects or alters the material's microstructure.

The series resistance decreases from 25 Ω, with annealing at 140 °C, to 20 Ω at 150 °C, indicating a significant improvement in electrical conduction and charge transport efficiency, associated with a more favorable morphological reorganization of the material. However, when the annealing temperature is raised to 160 °C, there is an increase in R_s to 28 Ω, suggesting that excessively high temperatures can induce undesirable structural changes, such as partial degradation of the film or an increase in defects that limit the mobility of charge carriers. The shunt resistance, on the other hand, behaves inversely, with a progressive increase in R_{sh} as the annealing temperature is increased, from 1017 Ω (140°C) to 1477 Ω (150°C) and reaching 3493 Ω at 160°C. This increase is indicative of a more effective suppression of leakage current paths, pointing to an improvement in the quality of the electrode/active film interface and in the uniformity of the coating, which contributes to the mitigation of internal short circuits and losses due to unwanted recombination. In addition, high R_{sh} values are directly associated with the reduction of parallel current paths that favor non-radiative recombination mechanisms, often related to trap states at the interface or in the volume of the active layer.

These observations highlight the critical role of precise thermal control during the fabrication process of PEDOT:PSS-based devices. An optimal annealing temperature promotes stable device performance. In particular, maintaining annealing temperatures around 150 °C appears to achieve the ideal balance between phase separation, crystallinity, defect minimization, and the maximization of parameters such as PCE.

S2 Theoretical Calculation Details

We employed Density Functional Theory (DFT) to investigate the key properties of PEDOT:PSS in interaction with NaCl. All DFT calculations were performed using the Gaussian 16 software package.[9] Geometry optimization to determine the lowest-energy configuration was conducted with the long-range corrected ωB97XD functional,[10] which accounts for both short- and long-range interactions, in conjunction with the 6-31G(d,p) basis set.[11,12]

The Gibbs free energy was computed at the same level of theory, ωB97XD/6-31G(d,p), by analyzing the vibrational modes of the isolated PEDOT, PSS, and NaCl molecules. Subsequently, the interaction between PEDOT:PSS and NaCl was examined in two distinct configurations: Configuration 1, where NaCl is positioned closest to PEDOT, and Configuration 2, where NaCl is placed closest to PSS. These configurations are illustrated in Figure S3.

S3 Analysis of Chemical Bond Lengths, PEDOT Chain Length, and Thiophene Ring Dihedral Angles

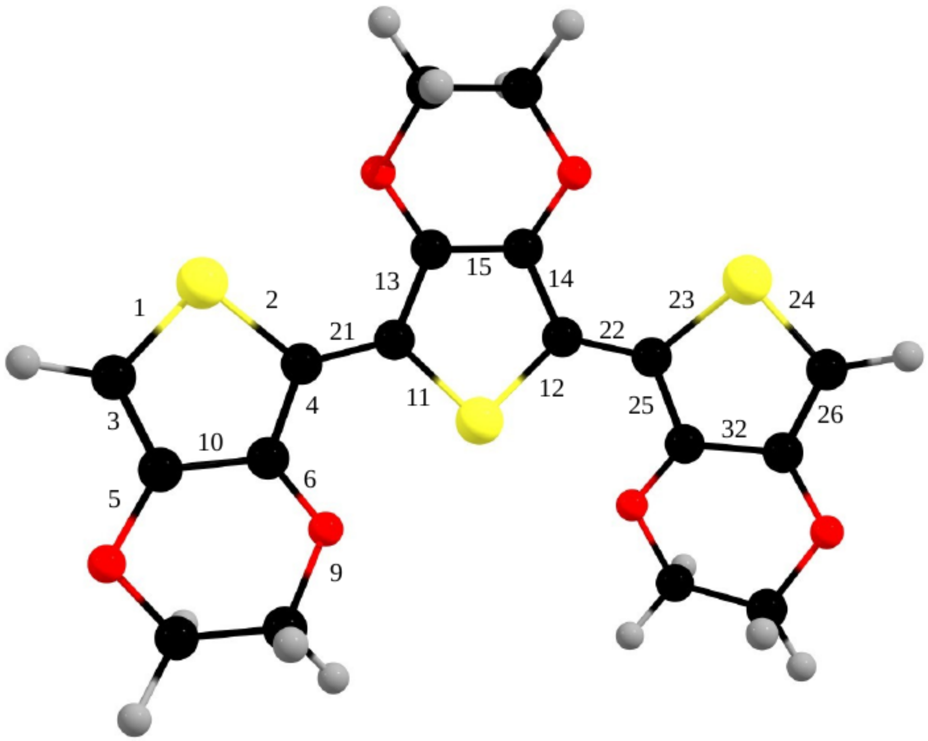

Figure S3: Visualization of the PEDOT structure and the chemical bonds analyzed in Table S2. The numbers correspond to the bond indices.

Table S2: Chemical bond lengths in PEDOT. The parameter ζ represents the ratio between the original bond length and the bond length after interaction with NaCl in Configuration 1 and Configuration 2.

Bond Index	PEDOT	Configuration 1	Configuration 2	ζ_{Config1}	ζ_{Config2}
1	1.7174	1.7160	1.7816	1.0000	0.9639
2	1.7598	1.7645	1.7457	0.9973	1.0080
3	1.3696	1.3653	1.3322	1.0031	1.0280
4	1.4154	1.4102	1.5094	1.0036	0.9377
5	1.3470	-	1.3439	-	-
6	1.3083	-	1.3439	-	-
9	1.4529	-	1.4421	-	-
10	1.4129	1.4129	1.4986	1.0000	0.9428
11	1.7548	1.7410	1.7723	1.0079	0.9901
12	1.7604	1.8494	1.7452	0.9518	1.0087
13	1.4339	1.4288	1.4344	1.0035	0.9996
14	1.4395	1.4939	1.4019	0.9635	1.0268
15	1.3607	1.4939	1.4019	0.9635	1.0268
21	1.3777	1.3811	1.3523	0.9975	1.0187
22	1.3710	1.4730	1.4055	0.9307	0.9754
23	1.7632	1.7403	1.7518	1.0131	1.0065
24	1.7201	1.7287	1.7180	0.9950	1.0012
25	1.4218	1.3705	1.3938	1.0374	1.0200
26	1.3693	1.3628	1.3656	1.0047	1.0027
32	1.4142	1.4284	1.4183	0.9900	0.9971

PEDOT

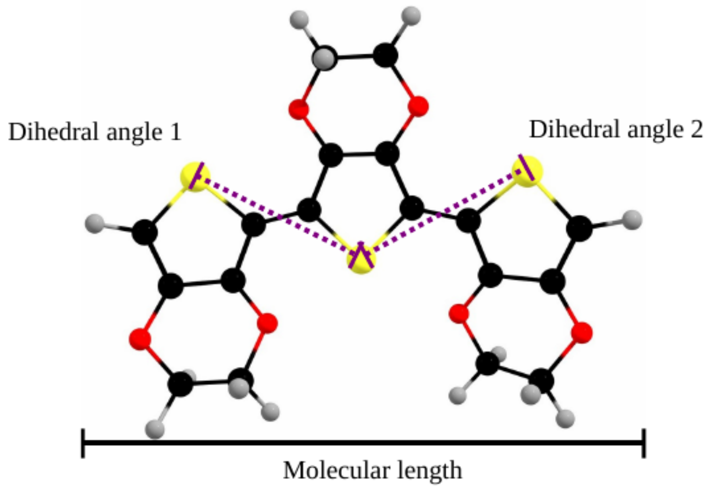

Dihedral angle or Molecular length	Molecular length (Å)	Dihedral angle 1 (Å)	Dihedral angle 2 (Å)
PEDOT	11.894	173.432	165.212
TESTE 1	12.172	161.171	150.189
TESTE 2	12.258	173.668	177.157

Figure S4: Analysis of PEDOT chain length and the dihedral angles between thiophene rings. The chain length was measured before and after interaction with NaCl.

S4 Analysis of HOMO and LUMO Molecular Orbitals

Tests 1 and 2 correspond to simulations of the PEDOT:PSS system in the presence of NaCl. In Test 1, the NaCl was initially positioned closer to the PEDOT segment, while in Test 2, it was placed nearer to the PSS. However, after structural optimization, it was observed that in both cases the NaCl tends to interact preferentially with PEDOT, even when initially positioned closer to PSS.

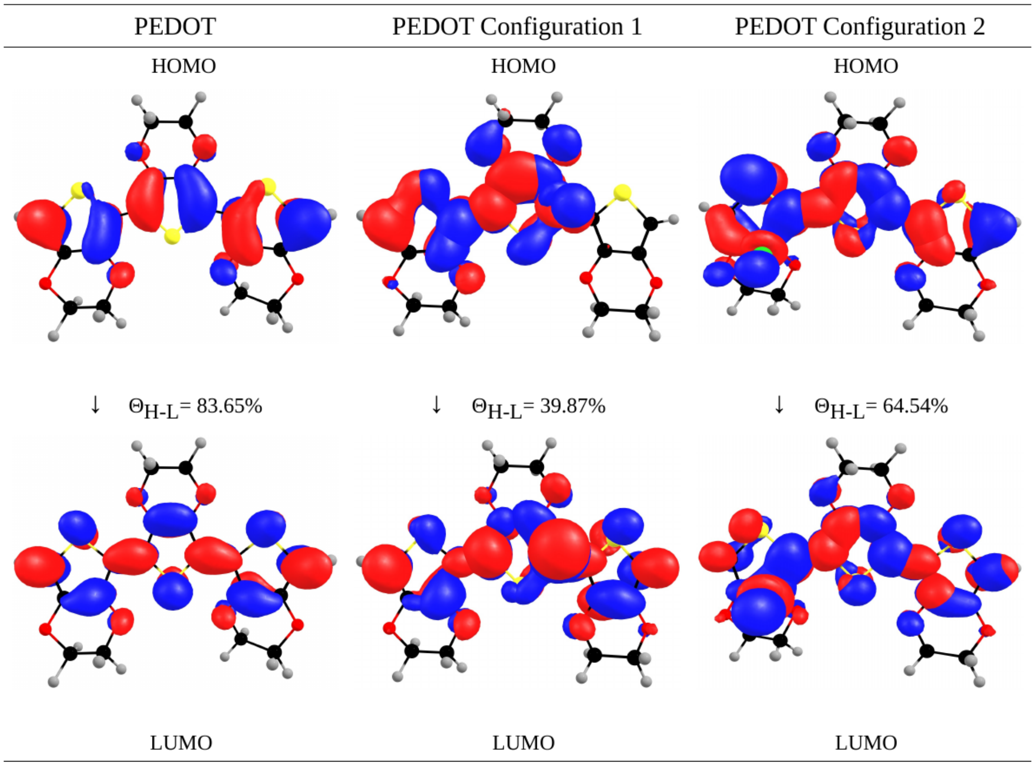

Figure S5: Visualization of the Highest Occupied Molecular Orbital (HOMO) and the Lowest Unoccupied Molecular Orbital (LUMO) of PEDOT. The analysis compares the molecular orbitals before and after interaction with NaCl, highlighting changes in electronic distribution. The Θ_{H-L} is the percentage value of the overlap of the HOMO and LUMO orbitals.

S5 Comparison between the electrical parameters of different samples of PEDOT:PSS doped with NaCl at various concentrations

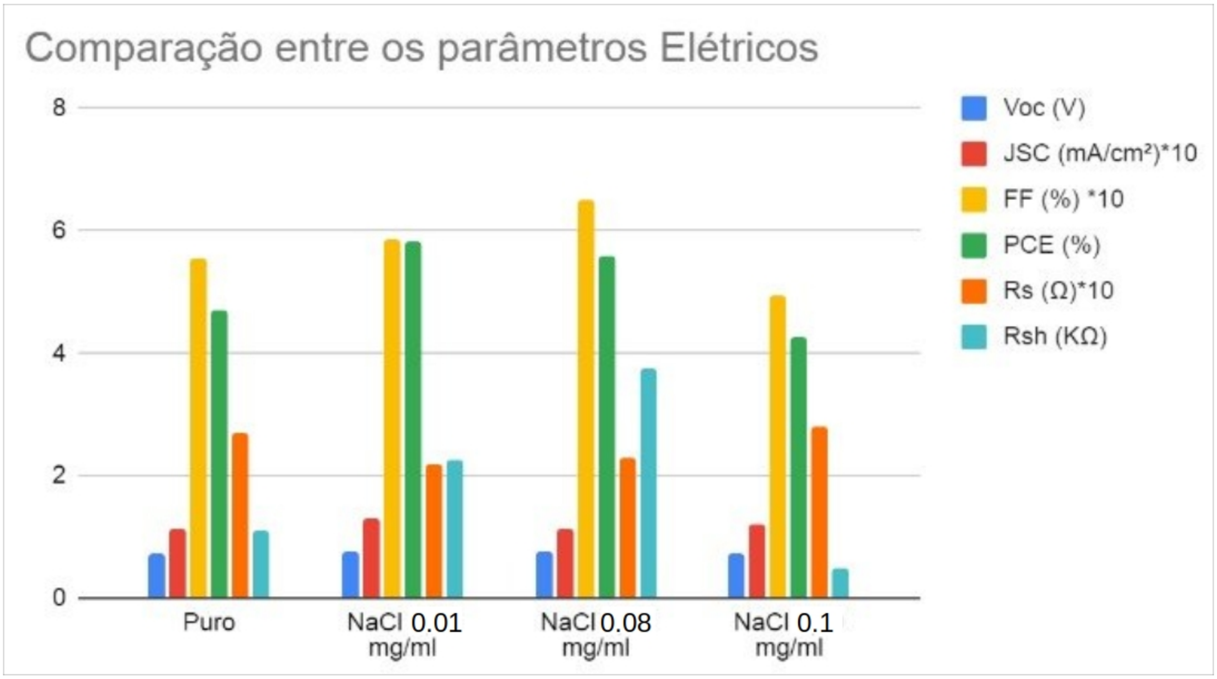

Figure S6: Electrical parameters of different samples of PEDOT:PSS doped with NaCl at various concentrations. The parameters presented include Voc (Open Circuit Voltage), JSC (Short Circuit Current Density), FF (Fill Factor), PCE (power conversion efficiency), Rs (Series Resistance), and Rsh (Shunt Resistance). The colored bars represent the values of each parameter for the Pure, NaCl 0.01 mg/ml, NaCl 0.08 mg/ml, and NaCl 0.10 mg/ml samples.

S6 Chemical structure of the donor polymer PV2001

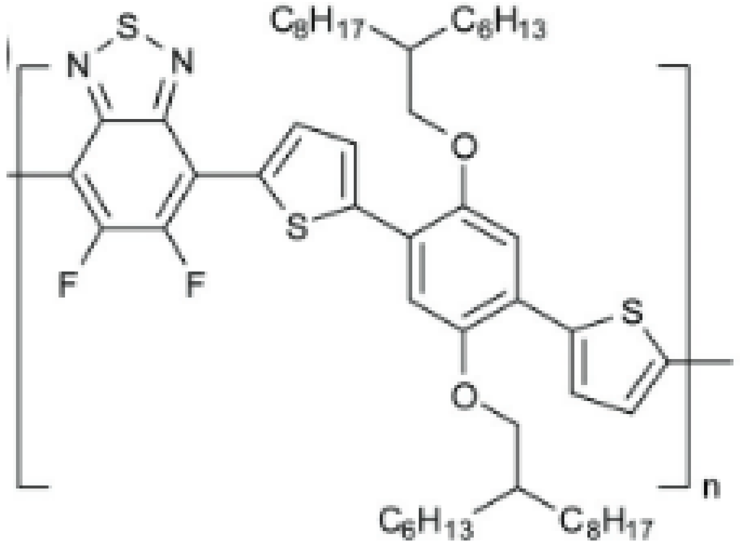

Figure S7: Chemical structure of the donor polymer PV2001.

References

(1) Watts, B.; Belcher, W. J.; Thomsen, L.; Ade, H.; Dastoor, P. C. A quantitative study of PCBM diffusion during annealing of P3HT: PCBM blend films. *Macromolecules* **2009**, *42*, 8392–8397.

(2) Verploegen, E.; Miller, C. E.; Schmidt, K.; Bao, Z.; Toney, M. F. Manipulating the morphology of P3HT–PCBM bulk heterojunction blends with solvent vapor annealing. *Chemistry of Materials* **2012**, *24*, 3923–3931.

(3) Wang, T.; Pearson, A. J.; Dunbar, A. D.; Staniec, P. A.; Watters, D. C.; Yi, H.; Ryan, A. J.; Jones, R. A.; Iraqi, A.; Lidzey, D. G. Correlating structure with function in thermally annealed PCDTBT: PC70BM photovoltaic blends. *Advanced Functional Materials* **2012**, *22*, 1399–1408.

(4) Wang, T.; Pearson, A. J.; Lidzey, D. G.; Jones, R. A. Evolution of structure, optoelectronic properties, and device performance of polythiophene: fullerene solar cells during thermal annealing. *Advanced Functional Materials* **2011**, *21*, 1383–1390.

(5) Verploegen, E.; Mondal, R.; Bettinger, C. J.; Sok, S.; Toney, M. F.; Bao, Z. Effects of thermal annealing upon the morphology of polymer–fullerene blends. *Advanced Functional Materials* **2010**, *20*, 3519–3529.

(6) Agostinelli, T.; Lilliu, S.; Labram, J. G.; Campoy-Quiles, M.; Hampton, M.; Pires, E.; Rawle, J.; Bikondoa, O.; Bradley, D. D.; Anthopoulos, T. D.; others Real-time investigation of crystallization and phase-segregation dynamics in P3HT: PCBM solar cells during thermal annealing. *Advanced Functional Materials* **2011**, *21*, 1701–1708.

(7) Candan, İ. Enhancement of inverted organic solar cell parameters by post-production annealing process. *Semiconductor Science and Technology* **2021**, *36*, 115008.

(8) Alam, S.; Petoukhoff, C. E.; Jurado, J. P.; Aldosari, H.; Jiang, X.; Váry, T.; Al Nasser, H.; Dahman, A.; Althobaiti, W.; Lopez, S. P. G.; others Influence of thermal annealing on microstructure, energetic landscape and device performance of P3HT: PCBM-based organic solar cells. *Journal of Physics: Energy* **2024**, *6*, 025013.

(9) Frisch, M. e.; Trucks, G.; Schlegel, H. B.; Scuseria, G.; Robb, M.; Cheeseman, J.; Scalmani, G.; Barone, V.; Petersson, G.; Nakatsuji, H.; others Gaussian 16. 2016.

(10) Chai, J.-D.; Head-Gordon, M. Long-range corrected hybrid density functionals with damped atom–atom dispersion corrections. *Physical Chemistry Chemical Physics* **2008**, *10*, 6615–6620.

(11) Zhao, W.; Qian, D.; Zhang, S.; Li, S.; Inganäs, O.; Gao, F.; Hou, J. Fullerene-Free Polymer Solar Cells with over 11% Efficiency and Excellent Thermal Stability. *Advanced Materials (Deerfield Beach, Fla.)* **2016**, *28*, 4734–4739.

(12) Zheng, Z.; Bredas, J.-L.; Coropceanu, V. Description of the charge transfer states at the pentacene/C60 interface: Combining range-separated hybrid functionals with the polarizable continuum model. *The Journal of Physical Chemistry Letters* **2016**, *7*, 2616–2621.
